# Extracranial carotid plaque calcification and its association with risk factors for cerebrovascular events: insights from the ANTIQUE study

**DOI:** 10.3389/fneur.2025.1532883

**Published:** 2025-01-29

**Authors:** David Pakizer, Dana Šalounová, David Školoudík

**Affiliations:** Centre for Health Research, Department of Clinical Neurosciences, Faculty of Medicine, University of Ostrava, Ostrava, Czechia

**Keywords:** atherosclerosis, carotid artery disease, calcification, cerebrovascular disease, magnetic resonance imaging, computed tomography

## Abstract

**Introduction:**

Extracranial carotid calcification is a common marker of advanced atherosclerosis. However, its impact on stroke risk is not consistent across studies, and examining the type of calcification and the presence of systemic diseases might be helpful. We aimed to investigate extracranial carotid calcification and its association with risk factors for ischemic cerebrovascular diseases.

**Materials and methods:**

Among 1,863 consecutive patients in the Atherosclerotic Plaque Characteristics Associated with a Progression Rate of the Plaque and a Risk of Stroke in Patients with the Carotid Bifurcation Plaque Study (ANTIQUE), 132 symptomatic or asymptomatic patients (177 carotid plaques) with >30% carotid stenosis examined through computed tomography (CT) and magnetic resonance imaging (MRI) were included. Statistical data were assessed using the *χ*^2^-test, Fisher’s exact test, *t*-test, and Mann–Whitney test to investigate the calcification risk factors.

**Results:**

Compared to the absence of calcifications, spotty calcifications were associated with male sex [odds ratio (OR): 3.72, 95% confidence interval (CI): 1.06–13.05], while large calcifications were associated with older patients (OR: 1.60 per 5 years of age, 95% CI: 1.20–2.13). Large calcifications were also strongly associated with coronary heart disease (OR: 4.07, 95% CI: 1.15–14.44) and atrial fibrillation (*p* = 0.025). In comparison between only spotty and large calcifications, spotty calcifications were associated with male sex (OR: 3.72, 95% CI: 1.06–13.05), smoking (*p* = 0.020) in more significant quantities (*p* = 0.014), and lipid plaque (*p* < 0.001), while large calcifications with contralateral stenosis degree (*p* = 0.044). No significant relationship was found between cerebrovascular events and the type of calcification.

**Conclusion:**

Although the presence and type of extracranial carotid calcification were not related to ipsilateral ischemic events, large calcifications were strongly associated with coronary heart disease and atrial fibrillation.

**Clinical trial registration:**

ClinicalTrials.gov, identifier NCT02360137.

## 1 Introduction

Carotid artery stenosis caused by atherosclerosis represents a substantial global epidemiological burden, with the prevalence of carotid plaque at 21% among individuals aged 30–79 years (1). Importantly, carotid atherosclerosis accounts for up to 25% of all ischemic strokes, which are among the leading causes of mortality and disability worldwide (2, 3). Extracranial carotid calcification represents a well-known clinical marker of atherosclerosis, which is characteristic of arterial aging. It is present in up to 75% of the population over the age of 75 years (1, 4).

The immediate inflammatory response that results in microcalcifications represents the pathophysiology of carotid calcification, which can be detected by non-invasive imaging methods, such as computed tomography (CT) and magnetic resonance imaging (MRI) (5). While microcalcifications and spotty calcifications indicate active vascular calcification related to inflammation, causing plaque instability, macrocalcification is strongly inversely related to macrophage infiltration, causing plaque stabilization (6). However, the results of the previous studies are not consistent. Several comprehensive studies have shown that carotid plaque calcification is a protective plaque characteristic not associated with stroke (7–9), but some studies found a positive association between calcification and stroke (10–12). Therefore, examining the relationship between extracranial carotid calcification and systemic diseases that play a role in stroke risk might be useful for risk stratification in patients (13). To the best of our knowledge, when considering the calcification type and multiple atherosclerosis-related systematic diseases, there exists a lack of evidence of these types of relationships.

This study aimed to assess the association between extracranial carotid plaque calcification and risk factors for ischemic cerebrovascular diseases in both symptomatic and asymptomatic patients.

## 2 Materials and methods

### 2.1 Study design

This study presents a *post hoc* analysis of data from the prospective multicenter observational and cross-sectional Atherosclerotic Plaque Characteristics Associated with a Progression Rate of the Plaque and a Risk of Stroke in Patients with the Carotid Bifurcation Plaque Study (ANTIQUE; ClinicalTrials.gov Identifier: NCT02360137).

### 2.2 Sample characterization

For the present study, we enrolled all consecutive patients from the ANTIQUE study who has carotid stenosis of at least 30% and underwent clinical and diagnostic (CT and MRI) examinations. The patients were recruited into the comprehensive stroke center between October 2016 and March 2019 from those indicated for neurosonology examination in stroke prevention or acute stroke diagnostics (14, 15). The inclusion criteria were as follows: Patients aged above 30 years; atherosclerotic plaque in the carotid bifurcation or the proximal part of the internal carotid artery with a thickness of ≥2 mm in the transverse plane of the ultrasound B-mode measurement; calcification detected in the mentioned area of carotid bifurcation in CT examination; sufficient image quality from CT and MRI examinations; and patient self-sufficiency (modified Rankin scale score, 0–2 points). A carotid plaque, representing the most stenotic lesion when multiple plaques were present, causing stenosis at least 30% on ultrasound B-mode (transition from laminar to turbulent blood flow) was included and further assessed (16).

The exclusion criteria were as follows: Patients whose CT or MRI of the neck was not performed; insufficient CT and MR image quality of the patients; non-cooperative patients for the examinations; patients detected with carotid artery occlusion; patients undergoing stenting in the carotid bifurcation; and patients after invasive treatment of ipsilateral carotid artery (carotid endarterectomy or stenting).

Symptomatic patients were characterized as those with clinical signs of recent ipsilateral cerebrovascular ischemic events [transient ischemic attack (TIA), stroke, amaurosis fugax, and/or retinal infarction] in the last 90 days (time from symptom onset to imaging), excluding patients with other potential stroke etiologies (cardioembolic, lacunar, arterial dissection, vasculitis, other rare causes of stroke) (17). Both arteries from symptomatic patients were included: the artery ipsilateral to the cerebrovascular event (symptomatic) and the contralateral (asymptomatic). Patients without clinical signs of TIA/stroke in the relevant arterial territory within the last 90 days were classified as asymptomatic.

All patients were examined through CT (first-line modality—as soon as possible after the onset of symptoms or within 30 days of recruitment from the neurosonology laboratory for asymptomatic patients) and MRI within 7 days following the CT examination.

### 2.3 Computed tomography

All patients were examined by a standard multidetector CT angiography (CTA) of carotid and brain arteries using various machines, with an intravenous iodine contrast agent Iomeron^®^ 400 (Bracco Imaging, Milan, Italy) or Ultravist^®^ 370 (Bayer HealthCare Pharmaceuticals LLC, Berlin, Germany) administered with 50–100-mL doses. Multiplanar axial plane reconstructions (<1-mm slices) and sagittal and coronal maximum intensity projection reconstructions (3–8 mm) were assessed with a uniform window width and center of 700 and 200 Hounsfield units (HU), respectively.

Carotid artery stenosis severity was measured based on the North American Symptomatic Carotid Endarterectomy Trial (NASCET) criteria (18). Plaque morphology was analyzed using density measurement of individual characteristics in HU. Characteristics were classified as lipid (<60 HU), fibrous (60–130 HU), or calcified (>130 HU) based on voxel-level measurements within regions of interest (2–10 pixels per region, covering a minimum of three plaque slices) (19). For overall plaque evaluation (lipid, fibrous, or calcified), the predominant characteristic had to occur in >50% plaque area. Calcifications were divided according to size into spotty (<3 mm in length/width) or large (>3 mm) (20). Additionally, smooth (no irregularities), irregular (minor surface changes), or ulcerated (>1 mm deep excavation in at least two planes) plaque surface was evaluated (19).

### 2.4 Magnetic resonance imaging

Carotid MRI examination protocol was conducted on different 1.5-Tesla machines with head/neck coil, consisting of the following sequences: Fat-suppressed T1-weighted_TSE [turbo spin echo; echo time (TE) 19 ms, repetition time (TR) 600 ms; slice thickness 3 mm; duration 3:50 min], 3D_T1-weighted_MPRAGE (magnetization prepared rapid gradient echo; TE 4 ms; TR 670 ms; inversion time 370 ms; 1 mm; 5:49 min), T2-weighted TSE (TE 72 ms; TR 4,580 ms; 4 mm; 3:18 min), and 3D_TOF (time of flight; TE 7 ms; TR 24 ms; 1 mm; 2:43 min).

In the individual plaque characteristics evaluation, differently distributed intraplaque signal intensities were visually compared to sternocleidomastoid muscle intensity. Overall, plaque composition was evaluated by the modified American Heart Association (AHA) plaque classification for MRI (IV–V, VI: unstable soft plaques; VII, VIII: stable hard plaques) (21). Lipid-rich necrotic core (LRNC; TOF: isointense, T1-w: isointense to hyperintense, T2-w: hypointense) and LRNC covering fibrous cap status (thick, thin, or ruptured) were assessed (22). Finally, intraplaque hemorrhage (IPH) categorized into acute (<1 week old; T1-w, TOF: hyperintense, T2-w: iso to hypointense) and subacute (1–6 weeks old; T1-w, T2-w, TOF: hyperintense) was evaluated.

All mentioned CT- and MRI-derived carotid plaque characteristics were evaluated by a single experienced rater (D.P.), blinded to patient medical history and CT results, based on cited major studies and expert consensus (5).

### 2.5 Demographic and clinical patient data

From the patient anamnestic data, the following atherosclerosis-related risk factors were retrieved: sex, age, arterial hypertension, diabetes mellitus, hyperlipidemia, bronchial asthma, chronic obstructive pulmonary disease, nephropathy, hyperuricemia, cancer, smoking, and alcohol. Daily cigarette and alcohol consumption (1 unit/20 g of alcohol = beer 0.5 L or wine 0.2 L or spirits 0.05 L) in the last year was also recorded. Moreover, data regarding atherosclerosis-related diseases (coronary heart disease, myocardial infarction, atrial fibrillation, and peripheral arterial disease) and cerebrovascular events (ischemic stroke, hemorrhagic stroke, transient ischemic attack, amaurosis fugax, and retinal infarction) were collected.

### 2.6 Statistical analysis

A statistical study power calculation was carried out. For a medium effect size *w* = 0.3, the significance level 0.05, and the test power 0.8 in the 2 × 2 table, the total sample size equal to 88 was sufficient. To account for the low quality of data in 25%, 110 patients were considered as a minimum to be recruited for the study.

The baseline characteristics were analyzed using descriptive statistics. Continuous data were noted as means ± standard deviations (SD) or medians and ranges. The categorical data were presented as numbers and percentages. Baseline differences between asymptomatic and symptomatic arteries were analyzed using the *χ*^2^-test of independence for contingency tables for categorical variables. If the assumption that the value of the expected cell counts is 5, or more, in at least 80% of the cells, and no cell has an expected count less than one was violated, Fisher’s exact test was used. Differences in continuous variables were assessed using the independent samples’ *t*-test for normally distributed variables or Mann–Whitney test otherwise. The normality of data was evaluated through the Shapiro–Wilk test.

Associations between the mentioned risk factors and calcification type (spotty, large) were assessed using the *χ*^2^-test, Fisher’s exact test, *t*-test, or Mann–Whitney test. Relationships between calcification type (spotty and large) and other plaque characteristics (CT: plaque type, plaque surface; MRI: AHA type, LRNC, fibrous cap, IPH), side of stenosis (ipsilateral, contralateral) were assessed using *χ*^2^-test or Fisher’s exact test with *post hoc* comparisons using adjusted residuals, or Mann–Whitney test. Associations of calcification type (no calcification, spotty, and large) and atherosclerosis-related diseases were evaluated by a *χ*^2^-test or Fisher’s exact test with *post hoc* comparisons using adjusted residuals. Detailed tables of the adjusted residuals are provided in Supplementary Tables S1–S5).

As a direct outcome, relationships between the calcification type (none, spotty, and large) and mentioned cerebrovascular events were assessed via the *χ*^2^-test or Fisher’s exact test where appropriate. Statistical significance was assumed at a *p*-value of <0.05. All analyses were performed using IBM-SPSS Statistics version 29.0 for Windows.

## 3 Results

### 3.1 Study population

**Figure 1:**
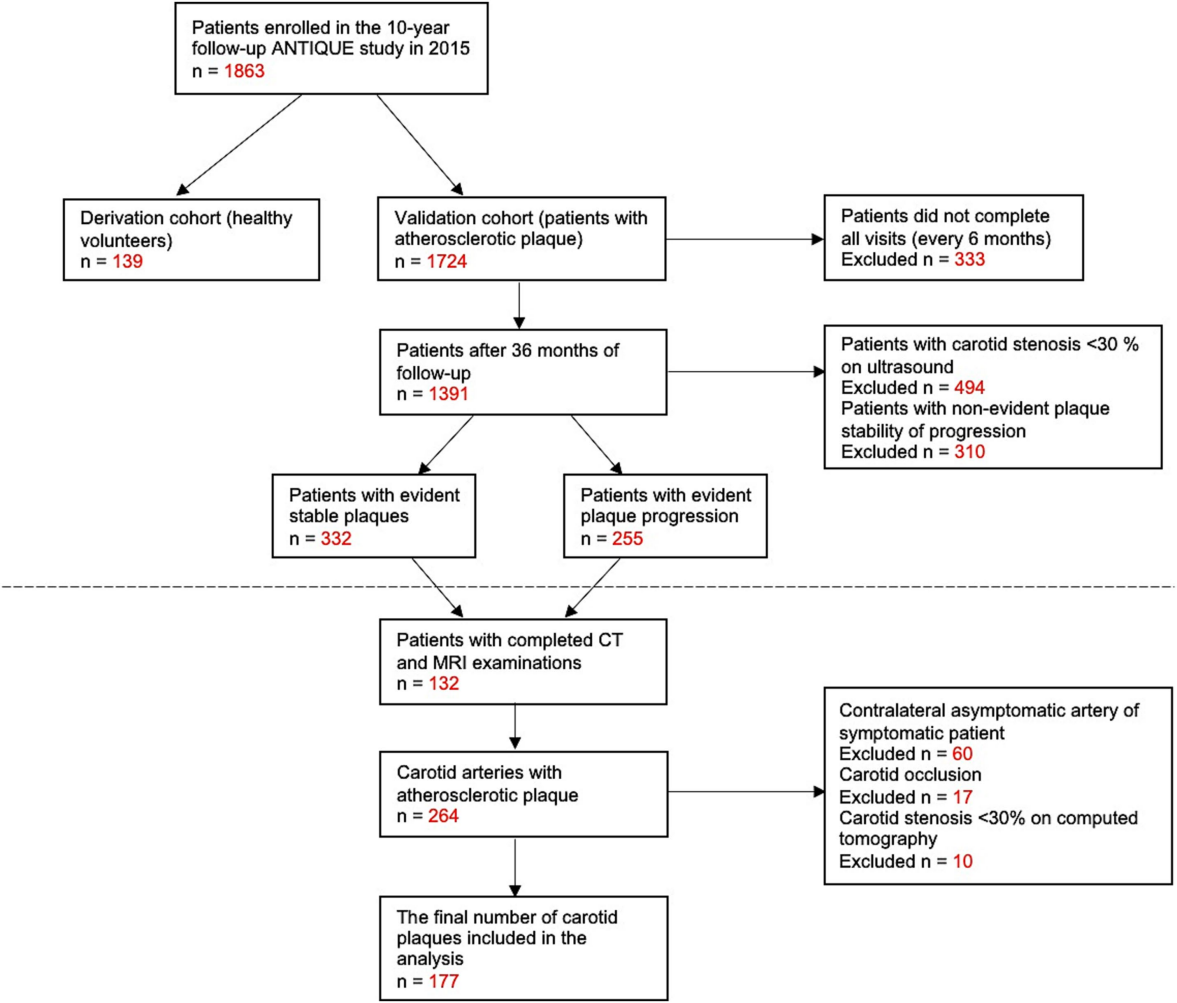
Study flowchart diagram.

Overall, 132 patients (264 carotid bifurcations) were examined by CT and MRI from 1,863 patients enrolled in the ANTIQUE study. Only a symptomatic artery was included from symptomatic patients (not the contralateral asymptomatic artery) to reach homogeneous groups of plaques. From 264 carotid arteries, 177 plaques (68.9% male individuals; median age of 69 years) were included, and 87 arteries were excluded due to asymptomatic artery of symptomatic patient (60 cases), carotid occlusion (17 cases), and carotid stenosis <30% (10 cases). The study flow chart is presented in Figure 1. Symptomatic patients were significantly more likely to consume alcohol in larger quantities, have hyperuricemia, and have less frequent coronary heart disease compared to asymptomatic patients. All the details about the study population are available in Table 1.

**Table 1:** Baseline patient characteristics.

	Patients included in the study (*n* = 132)	Arteries included in analyses		
		All arteries (*n* = 177)	Only asymptomatic arteries (*n* = 117)	Only symptomatic arteries (*n* = 60)
Male sex	93 (70.5)	122 (68.9)	79 (67.5)	43 (71.7)
Patient age (years); median (range) in years	68.5 (44–88)	69 (44–88)	69 (49–86)	68 (44–88)
Atherosclerosis risk factors				
Hypertension	121 (91.7)	164 (92.7)	111 (94.9)	53 (88.3)
Diabetes mellitus	55 (41.7)	75 (42.4)	50 (42.7)	25 (41.7)
Hyperlipidemia	98 (74.2)	133 (75.1)	86 (73.5)	47 (78.3)
Smoking	44 (33.3)	59 (33.3)	35 (29.9)	24 (40)
Number of cigarettes per day; median (range)	0 (0–40)	0 (0–40)	0 (0–40)	0 (0–40)
Alcohol	51 (38.6)	62 (35.0)	28 (23.9)	34 (56.7)^a^
Number of alcohol units per day; median (range)	0.0 (0–3)	0.0 (0–3)	0.0 (0–3)	1.0 (0–3)^c^
Chronic diseases				
Bronchial asthma	2 (1.5)	3 (1.7)	2 (1.7)	1 (1.7)
Obstructive pulmonary disease	10 (7.6)	13 (7.3)	10 (8.5)	3 (5)
Nephropathy	9 (6.8)	10 (5.6)	5 (4.3)	5 (8.3)
Hyperuricemia	11 (8.3)	14 (7.9)	4 (3.4)	10 (16.7)^b^
Cancer	4 (3)	4 (2.3)	1 (0.9)	3 (5)
Atherosclerosis-related diseases				
Coronary heart disease	40 (30.3)	56 (31.6)	43 (36.8)	13 (21.7)^a^
Myocardial infarction	24 (18.2)	32 (18.1)	25 (21.4)	7 (11.7)
Atrial fibrillation	18 (13.6)	24 (13.6)	15 (12.8)	9 (15)
Peripheral arterial disease	20 (15.2)	25 (14.1)	16 (13.7)	9 (15)
Cerebrovascular events				
Ischemic stroke	36 (27.3)	37 (20.9)	0 (0)	37 (61.7)^a^
Hemorrhagic stroke	1 (0.8)	2 (1.1)	0 (0)	2 (3.3)
Transient ischemic attack	13 (9.8)	15 (8.5)	0 (0)	15 (25)^a^
Amaurosis fugax	4 (3)	4 (2.3)	0 (0)	4 (6.7)^b^
Retinal infarction	2 (1.5)	2 (1.1)	0 (0)	2 (3.3)
Extracranial calcifications				
Absent		24 (13.6)	13 (11.1)	11 (18.3)
Spotty only		36 (20.3)	25 (21.4)	11 (18.3)
Large		117 (66.1)	79 (67.5)	38 (63.3)
Plaque type				
Lipid		89 (50.3)	53 (45.3)	36 (60)
Fibrous		47 (26.6)	35 (29.9)	12 (20)
Calcified		41 (23.2)	29 (24.8)	12 (20)

All data are presented as “*n* (%),” if not specified differently.

a Significant difference between asymptomatic and symptomatic patients at the level *p* < 0.05, analyzed using the *χ*^2^-test.

b Significant difference between asymptomatic and symptomatic patients at the level *p* < 0.05, analyzed using Fisher’s exact test.

c Significant difference between asymptomatic and symptomatic patients at the level *p* < 0.05, analyzed using the Mann–Whitney test.

### 3.2 Risk factors for the presence of different calcification types

When assessing the risk factors regarding absent calcification, spotty calcifications were associated with male sex [crude odds ratio (OR) 3.72, 95% confidence interval (CI) 1.06–13.05; Supplementary Table S6]. For large calcification, logistic regression analysis showed that these patients were significantly older (crude OR 1.60 per 5 years of age, 95% CI 1.20–2.13; Supplementary Table S6). Detailed results are presented in Supplementary Table S6 (crude ORs) and Supplementary Table S7 (adjusted ORs).

In comparison between only spotty and large calcifications, men had more often spotty calcifications, while women had more frequent large calcifications (*p* = 0.015). Higher age was associated with the presence of large calcification (*p* = 0.027). Smoking in more significant quantities was significantly related to spotty calcification (*p* = 0.014). At the same time, alcohol consumption, along with other atherosclerosis risk factors and chronic diseases, did not differ between the groups. All the above-mentioned results are presented in Table 2.

**Table 2:** Risk factors for extracranial carotid plaque calcifications.

	Absent (*n* = 24)	Spotty calcification only (*n* = 36)	Large calcification (*n* = 117)	*p*-value^a^
Male sex	15 (62.5)	31 (86.1)	76 (65)	0.015^b^
Patient age; mean ± SD (years)	65.0 ± 9.8	68.2 ± 7.9	71.5 ± 7.9	0.027^d^
Atherosclerosis risk factors				
Hypertension	21 (87.5)	35 (97.2)	108 (92.3)	0.45^c^
Diabetes mellitus	8 (33.3)	15 (41.7)	52 (44.4)	0.77^b^
Hyperlipidemia	17 (70.8)	30 (80.3)	86 (73.5)	0.23^b^
Smoking	7 (29.2)	18 (50)	34 (29.1)	0.020^b^
Number of cigarettes per day; median (range)	0 (0–30)	2.5 (0–40)	0 (0–30)	0.014^e^
Alcohol	9 (37.5)	11 (30.6)	42 (35.9)	0.56^b^
Number of alcohol units per day; median (range)	0.0 (0–2)	0.0 (0–2)	0.0 (0–3)	0.41^e^
Chronic disease				
Bronchial asthma	1 (4.2)	1 (2.8)	1 (0.9)	0.42^e^
Obstructive pulmonary disease	1 (4.2)	5 (13.9)	7 (6)	0.16^c^
Nephropathy	1 (4.2)	1 (2.8)	8 (6.8)	0.69^c^
Hyperuricemia	3 (12.5)	4 (11.1)	7 (6)	0.29^c^
Cancer	2 (8.3)	0 (0)	2 (1.7)	>0.99^c^

All data are presented as “*n* (%),” if not specified differently.

a *p-values are given for a difference between the groups with spotty calcifications only and large calcifications.*

b Analyzed using the *χ*^2^-test.

c Analyzed using Fisher’s exact test where appropriate.

d Analyzed using the *t*-test.

e Analyzed using the Mann–Whitney test.

### 3.3 Calcification and other plaque characteristics

The calcification type was not related to the degree of ipsilateral stenosis but to contralateral stenosis. Large calcification was associated with a high degree of stenosis contralaterally (*p* = 0.044). Lipid plaque on CT was associated with spotty calcification (*p* < 0.001), while a large calcification was associated with calcified plaque (*p* < 0.001). Spotty calcification showed a non-significant trend toward more frequent presence in plaques with irregular surfaces than large calcification (*p* = 0.16). No significant associations were found between calcification patterns and MRI-derived carotid plaque characteristics. However, non-significant trends suggested spotty calcifications were more common in AHA type IV–V plaques (LRNC surrounded by fibrous tissue with possible calcification), plaques with LRNC, and thin or ruptured fibrous cap; and large calcification were more common in AHA type VII (calcified plaque) and subacute IPH. Detailed results on CT- and MRI-derived plaque characteristics are presented in Table 3.

**Table 3:** Extracranial carotid plaque calcification and other plaque characteristics evaluated on computed tomography (CT) and magnetic resonance imaging (MRI).

	Spotty calcification only (*n* = 36)	Large calcification (*n* = 117)	*p*-value
Computed tomography			
Severity of ipsilateral stenosis; mean ± SD %	76.1 ± 15.1	71.4 ± 17.9	0.14^c^
Severity of contralateral stenosis; mean ± SD %	54.4 ± 33.3	68.0 ± 26.3	0.044^c^
Plaque type			
Lipid	26 (72.2)	47 (40.2)	<0.001^a^
Fibrous	9 (25)	30 (25.6)	
Calcified	1 (2.8)	40 (34.2)	
Surface			
Smooth	0 (0)	5 (4.3)	0.16^b^
Irregular	18 (50)	46 (39.3)	
Ulcerated	18 (50)	66 (56.4)	
Magnetic resonance imaging			
AHA plaque type			
IV–V	17 (56.7)	34 (37)	0.076^a^
VI	9 (30)	28 (30.4)	
VII	1 (3.3)	21 (22.8)	
VIII	3 (10)	9 (9.8)	
Lipid-rich necrotic core	26 (86.7)	60 (73.2)	0.13^a^
Intraplaque hemorrhage			
Acute	4 (12.5)	12 (11.1)	0.38^b^
Subacute	1 (3.1)	11 (10.2)	
Fibrous cap			
Thick	10 (33.3)	27 (32.1)	0.16^a^
Thin	7 (23.3)	12 (14.3)	
Ruptured	7 (23.3)	11 (13.1)	

All data are presented as “*n* (%),” if not specified differently. Note that in 17 plaques assessed through CT, the MRI quality was insufficient to analyze those plaques on MRI, resulting in 199 plaques evaluated by MRI and 216 by CT.

a Analyzed using a *χ*^2^-test.

b Analyzed using Fisher’s exact test.

c Analyzed using the Mann–Whitney test.

### 3.4 Calcification in atherosclerosis-related diseases and cerebrovascular events

No significant relation was found between ipsilateral cerebrovascular events and the presence or type of calcification. However, atrial fibrillation was significantly more often in patients with large calcification within carotid plaque (*p* = 0.015, overall test, Table 4). Large calcifications were associated with coronary heart disease (crude OR 4.07, 95% CI 1.15–14.44; Supplementary Table S8) and atrial fibrillation (*p* = 0.025; Supplementary Table S8) compared to no calcification. Further results are provided in Table 4, and crude and adjusted OR values are given in the Supplementary Tables S8, S9, respectively.

**Table 4:** Extracranial carotid plaque calcification in association with atherosclerosis-related diseases and cerebrovascular events.

	Absent (*n* = 24)	Spotty calcification only (*n* = 36)	Large calcification (*n* = 117)	*p*-value^a^
Coronary heart disease	3 (12.5)	10 (27.8)	43 (36.8)	0.057^b^
Myocardial infarction	2 (8.3)	6 (16.7)	24 (20.5)	0.36^b^
Atrial fibrillation	0 (0)	4 (11.1)	20 (17.1)	0.015^c^
Peripheral arterial disease	2 (8.3)	8 (22.2)	15 (12.8)	0.25^b^
Ischemic stroke	6 (25)	9 (25)	22 (18.8)	0.63^b^
Hemorrhagic stroke	0 (0)	0 (0)	2 (1.7)	0.43^c^
Transient ischemic attack	4 (16.7)	2 (5.6)	9 (7.7)	0.33^c^
Amaurosis fugax	1 (4.2)	0 (0)	3 (2.6)	0.37^c^
Retinal infarction	0 (0)	0 (0)	2 (1.7)	0.43^c^

All data are presented as “*n* (%)” if not specified differently. The numbers in the table do not sum to column totals, as patients could have had multiple conditions.

a *p-value for the overall test.*

b Analyzed using a *χ*^2^-test.

c Analyzed using Fisher’s exact test.

## 4 Discussion

We could not find an association between the presence and type of plaque calcifications and ipsilateral ischemic events (stroke, TIA, amaurosis fugax, or retinal infarction) in our study population. The presence of large carotid plaque calcification represented the highest association with coronary heart disease and atrial fibrillation, followed by higher patient age and female sex. On the other hand, spotty calcifications were associated with male sex, higher levels of smoking, and a greater prevalence in soft plaques.

While some evidence suggested a positive relationship between extracranial carotid calcification and ipsilateral ischemic events (10–12), particularly for spotty calcifications (23, 24), our study results are in agreement with the two recent comprehensive meta-analyses that identified negative association between carotid calcification and stroke (risk ratio: 0.75, OR: 0.5) (7, 9). In the interventional treatment, a large recent study found that a greater severity of carotid calcification (>50% of the plaque volume) is a significant risk factor for in-hospital stroke or death in 21,860 patients undergoing carotid artery stenting (25). Another study differentiated two calcium salts using dispersive X-ray microanalysis (hydroxyapatite, presented more in unstable plaque, and calcium oxalate, associated with plaque stability), suggesting different implications on plaque biology and subsequent stability (26). The distinction between these two types of calcium salts could have important clinical implications and can be investigated using dual-energy CT scanners to identify differences in tissue chemical composition (27, 28). Large calcifications relate with a gene transcriptional profile typical for stable plaques, repressed inflammation, and extracellular matrix organization (29). However, the association between spotty calcification and inflammatory markers, plaque instability, and accelerated disease progression should be noted (30). Finally, macrophages crucially control the mineralization process from microcalcification to bone-like tissue but are having accelerative and decelerative association with calcification. The bilateral interaction remains rather unexplored and should be studied (31).

Our study results proved the association between large calcification and generalized atherosclerosis manifested in a strong relationship with coronary heart disease, atrial fibrillation, and the severity of contralateral carotid stenosis. Two large population-based studies found the same results regarding the presence and extent of calcification and the risk of coronary heart disease (32, 33). However, a large meta-analysis revealed less prevalent carotid calcification in non-significant compared with significant coronary artery disease and moderate relation between carotid and coronary stenosis (34). Atherosclerosis affects both carotid and coronary systems, although not always in an identical phenotypic manner, so examination of carotid arteries is beneficial whenever coronary artery disease is suspected, mainly when large carotid calcification is detected. Despite the findings that patients with carotid atherosclerosis are at high risk of developing atrial fibrillation or both diseases coexist (35–37), no evidence of an association between carotid calcification and atrial fibrillation was found, which has been investigated in our study. Our positive risk association between large carotid calcification and atrial fibrillation was found only in a recent study but significantly after adjustments only in coronary plaques (38). The higher degree of contralateral carotid stenosis associated with carotid calcification demonstrated the presence of generalized atherosclerosis. However, possible overestimation of stenosis severity on CTA due to blooming artifacts from large carotid calcification should be considered (39).

Active smoking or exposure to cigarette smoke is responsible as a catalyst for the formation and development of unstable plaques (40). In particular, carotid calcification is promoted by nicotine (41), but no study was found with evidence of the influence of smoking on spotty calcification. In our study, only spotty calcifications were more often in smokers in greater quantities. The coexistence of spotty carotid calcifications and soft plaque characteristics (LRNC and IPH) (42, 43), typically associated with ipsilateral cerebrovascular events, is suggested in studies even in non-stenosing plaques (24). We found only an association between spotty calcification and lipid plaque but not with IPH or ischemic events. Although spotty calcifications might be at risk of stroke, meta-analyses confirmed that other carotid plaque characteristics are more associated with stroke (44, 45). Male sex was associated with carotid calcification compared to women (46), particularly when looking only at spotty calcification, similar to our study results (47). Calcification growth is mainly associated with increasing age, calcification load, hypertension, or smoking over time (48).

Additionally, extracranial calcification was associated with diabetes mellitus, hypertension (49), or hyperlipidemia (50) in previous studies, but we did not find any difference between them and spotty and large calcification in our study. Regarding the treatment of carotid calcification, high-density lipoprotein appears to benefit vascular calcification (51). Beneficial changes in serum calcification markers were found after ipsilateral carotid artery stenting with intensive lipid-lowering therapy to enhance contralateral carotid plaque stability in patients with bilateral carotid stenosis (52). However, no preferred treatment for extracranial carotid calcification is recommended by current guidelines. To our knowledge, this is the first study that complexly investigated the type of CT-derived extracranial carotid calcification associated with multiple atherosclerotic-related systematic diseases. Large or spotty plaque calcifications were not associated with cerebrovascular events, suggesting an association with plaque stability with no need for acute treatment. However, larger prospective studies and future efforts are warranted to study the effect of, particularly, carotid spotty calcifications on stroke risk.

This study has the following limitations. (1) Approximately 90% of all patients enrolled in the ANTIQUE study were excluded from our analysis due to stenosis degree >30% or mostly because of missing CT and MRI examination together because ultrasound was the first-line imaging modality accompanied by CT if needed or before invasive intervention (MRI underwent only a minority of patients). (2) Laboratory markers were not measured, as our primary focus was on the imaging-based presence of calcification and its relation with various atherosclerosis and stroke risk factors and other diseases. (3) Various CT and MRI devices were utilized due to the multicenter study design, which could introduce minor discrepancies in evaluating calcification and other plaque characteristics. Diagnostic modalities were calibrated using five plaques *in vitro* to minimize this variation.

## 5 Conclusion

Although the presence and type of extracranial carotid plaque calcification were not related to ipsilateral ischemic events, large calcification was strongly associated with coronary heart disease and atrial fibrillation. Higher levels of smoking was responsible for the presence of spotty calcification associated with male sex and the occurrence of soft plaques.

## Data Availability

The raw data supporting the conclusions of this article will be made available by the authors, without undue reservation.

## Group members for The ANTIQUE Study Group

David Netuka, Jiří Vrána, František Charvát (Military University Hospital Prague, Prague), Petra Kešnerová (University Hospital Motol, Prague); Tomáš Hrbáč, Tomáš Jonszta (University of Ostrava and University Hospital Ostrava, Ostrava); Roman Herzig (Charles University and University Hospital Hradec Králové, Hradec Králové).
