# Supplementary material for: Extracranial carotid plaque calcification and its association with risk factors for cerebrovascular events: insights from the ANTIQUE study
Source: Front Neurol. 2025 Jan 29;16:1532883. doi: 10.3389/fneur.2025.1532883 (PMC11813772; doi:10.3389/fneur.2025.1532883)
Supplement: Supplementary file 1 [file Data_Sheet_1.pdf]

Character of Extracranial Carotid Plaque Calcification and Its Association with Risk Factors of Cerebrovascular Events: Insights from the ANTIQUE Study

David Pakizer[a], Dana Šalounová[a], David Školoudík[a]; for The ANTIQUE Study Group

[a] Centre for Health Research, Department of Clinical Neurosciences, Faculty of Medicine, University of Ostrava, Ostrava, Czech Republic

Supplementary material

Supplementary tables

Table S1. Contingency table with adjusted residuals for calcification and plaque surface.

			Plaque surface			Total
			Smooth	Irregular	Ulcerated	
Calcification type	Spotty only	Count	0	18	18	36
		Adjusted residual	–1.3	1.1	–0.7	
	Large	Count	5	46	66	117
		Adjusted residual	1.3	–1.1	0.7	
Total		Count	5	64	84	153

Table S2. Contingency table with adjusted residuals for calcification and plaque type.

			Plaque type			Total
			Lipid	Fibrous	Calcified	
Calcification type	Spotty only	Count	26	9	1	36
		Adjusted residual	3.4	–0.1	–3.7	
	Large	Count	47	30	40	117
		Adjusted residual	–3.4	0.1	3.7	
Total		Count	73	39	41	153

Table S3. Contingency table with adjusted residuals for calcification and AHA plaques.

			General AHA plaque composition				Total
			IV–V	VI	VII	VIII	
Calcification type	Spotty only	Count	17	9	1	3	30
		Adjusted residual	1.9	0	–2.4	0	
	Large	Count	34	28	21	9	92
		Adjusted residual	–1.9	0	2.4	0	
Total		Count	51	37	37	12	122

AHA – American Heart Association

Table S4. Contingency table with adjusted residuals for calcification type and intraplaque hemorrhage.

<table>
<tr><th colspan="3" rowspan="2"></th><th colspan="3">Intraplaque hemorrhage type</th><th rowspan="2">Total</th></tr>
<tr><th>None</th><th>Acute</th><th>Subacute</th></tr>
<tr><td rowspan="4">Calcification type</td><td rowspan="2">Spotty only</td><td>Count</td><td>27</td><td>4</td><td>1</td><td rowspan="2">32</td></tr>
<tr><td>Adjusted residual</td><td>0.7</td><td>0.2</td><td>–1.3</td></tr>
<tr><td rowspan="2">Large</td><td>Count</td><td>85</td><td>12</td><td>11</td><td rowspan="2">108</td></tr>
<tr><td>Adjusted residual</td><td>–0.7</td><td>–0.2</td><td>1.3</td></tr>
<tr><td colspan="2">Total</td><td>Count</td><td>112</td><td>16</td><td>12</td><td>140</td></tr>
</table>

Table S5. Contingency table with adjusted residuals for calcification type and fibrous cap.

<table>
<tr><th colspan="3" rowspan="2"></th><th colspan="4">Fibrous cap</th><th rowspan="2">Total</th></tr>
<tr><th>None</th><th>Thick</th><th>Thin</th><th>Ruptured</th></tr>
<tr><td rowspan="4">Calcification type</td><td rowspan="2">Spotty only</td><td>Count</td><td>6</td><td>10</td><td>7</td><td>7</td><td rowspan="2">30</td></tr>
<tr><td>Adjusted residual</td><td>–2</td><td>0.1</td><td>1.1</td><td>1.3</td></tr>
<tr><td rowspan="2">Large</td><td>Count</td><td>34</td><td>27</td><td>12</td><td>11</td><td rowspan="2">84</td></tr>
<tr><td>Adjusted residual</td><td>2</td><td>–0.1</td><td>–1.1</td><td>–1.3</td></tr>
<tr><td colspan="2">Total</td><td>Count</td><td>40</td><td>37</td><td>19</td><td>18</td><td>114</td></tr>
</table>

Table S6. Risk factors for extracranial carotid plaque calcifications – crude odds ratios.

	**Spotty calcification only** OR (95%-CI)[a]	***p*-value**	**Large calcification** OR (95%-CI)[a]	***p*-value**
Male	**3.72 (1.06–13.05)**	**0.034**	1.11 (0.45–2.76)	0.819
Age (each five years)	1.24 (0.91–1.70)	0.170	**1.60 (1.20–2.13)**	**0.001**
Atherosclerosis risk factors				
Hypertension	5.00 (0.49–51.23)	0.292	1.71 (0.43–6.87)	0.430
Diabetes mellitus	1.43 (0.49–4.19)	0.515	1.60 (0.64–4.03)	0.316
Hyperlipidemia	2.06 (0.59–7.13)	0.250	1.14 (0.43–3.02)	0.788
Smoking	2.43 (0.81–7.27)	0.109	1.00 (0.38–2.62)	0.992
Alcohol	0.73 (0.25–2.18)	0.576	0.93 (0.39–2.32)	0.882
Chronic diseases				
Bronchial asthma	0.66 (0.04–11.04)	1.000	0.20 (0.01–3.29)	0.312
Obstructive pulmonary disease	3.71 (0.41–33.94)	0.387	1.46 (0.17–12.8)	1.000
Nephropathy	0.66 (0.04–11.04)	1.000	1.69 (0.20–14.16)	1.000
Hyperuricemia	0.88 (0.18–4.21)	1.000	0.45 (0.11–1.86)	0.374
Cancer	NA	0.156	0.19 (0.03–1.43)	0.134

OR = odds ratio, 95% CI = 95% confidence interval, [a] = compared to absent calcification.

Table S7. Risk factors for extracranial carotid plaque calcifications – odds ratios adjusted for age and sex computed using logistic regression.

	**Spotty calcification only** OR (95%-CI)[a]	***p*-value**	**Large calcification** OR (95%-CI)[a]	***p*-value**
Male	3.53 (0.99–12.59)	0.052	1.15 (0.44–2.99)	0.773
Age (each five years)	1.22 (0.88–1.69)	0.234	**1.60 (1.20–2.13)**	**0.001**
Atherosclerosis risk factors				
Hypertension	5.97 (0.52-68.21)	0.150	0.84 (0.18–3.91)	0.824
Diabetes mellitus	1.82 (0.57–5.83)	0.316	1.12 (0.42–3.01)	0.818
Hyperlipidemia	1.93 (0.49–7.56)	0.345	1.30 (0.46–3.68)	0.625
Smoking	2.63 (0.82–8.43)	0.105	1.33 (0.48–3.69)	0.583
Alcohol	0.79 (0.25–2.50)	0.684	0.96 (0.37–2.52)	0.940
Chronic diseases				
Bronchial asthma	1.18 (0.04–37.21)	0.925	0.42 (0.01–17.71)	0.652
Obstructive pulmonary disease	2.87 (0.29–28.28)	0.365	0.65 (0.07–6.05)	0.701
Nephropathy	0.60 (0.03–11.60)	0.738	0.53 (0.05–5.21)	0.587
Hyperuricemia	1.21 (0.21–6.96)	0.829	0.40 (0.08–1.93)	0.254
Cancer	NA	NA	0.12 (0.01–1.08)	0.058

OR = odds ratio, 95% CI = 95% confidence interval, [a] = compared to absent calcification.

Table S8. Extracranial carotid plaque calcification in association with atherosclerosis-related diseases and cerebrovascular events – crude odds ratios.

	**Spotty calcification only** OR (95%-CI)[a]	***p*-value**	**Large calcification** OR (95%-CI)[a]	***p*-value**
Coronary heart disease	2.69 (0.66–11.06)	0.159	**4.07 (1.15–14.44)**	**0.021**
Myocardial infarction	2.20 (0.41–11.95)	0.457	2.84 (0.62–12.92)	0.248
Atrial fibrillation	NA	0.143	**NA**	**0.025**
Peripheral arterial disease	3.14 (0.61-16.32)	0.289	1.62 (0.35–7.59)	0.737
Ischemic stroke	1.00 (0.30–3.30)	1.000	0.70 (0.25–1.95)	0.574
Hemorrhagic stroke	NA	NA	1.02 (0.99–1.04)	1.000
Transient ischemic attack	0.29 (0.05–1.75)	0.206	0.42 (0.12–1.49)	0.236
Amaurosis fugax	0.96 (0.88–1.04)	0.400	0.61 (0.06–6.08)	0.530
Retinal infarction	NA	NA	1.02 (0.99–1.04)	1.000

OR = odds ratio, 95%-CI = 95%-confidence interval, [a] = compared to absent calcification.

Table S9. Extracranial carotid plaque calcification in association with atherosclerosis-related diseases and cerebrovascular events – odds ratios adjusted for age and sex computed using logistic regression.

	**Spotty calcification only** OR (95%-CI)[a]	***p*-value**	**Large calcification** OR (95%-CI)[a]	***p*-value**
Coronary heart disease	2.55 (0.58–11.21)	0.214	2.77 (0.74–10.29)	0.129
Myocardial infarction	1.77 (0.30–10.35)	0.524	1.71 (0.35–8.31)	0.508
Atrial fibrillation	NA	NA	NA	NA
Peripheral arterial disease	2.56 (0.46–14.21)	0.282	0.81 (0.15–4.25)	0.804
Ischemic stroke	0.92 (0.26–3.26)	0.903	0.64 (0.21–1.91)	0.419
Hemorrhagic stroke	NA	NA	NA	NA
Transient ischemic attack	0.45 (0.07–3.14)	0.423	0.48 (0.12–1.95)	0.302
Amaurosis fugax	NA	NA	0.45 (0.04–5.28)	0.525
Retinal infarction	NA	NA	NA	NA

OR = odds ratio, 95%-CI = 95%-confidence interval, [a] = compared to absent calcification.
